# Synthesis, Property Characterization and Photocatalytic Activity of the Novel Composite Polymer Polyaniline/Bi_2_SnTiO_7_

**DOI:** 10.3390/molecules17032752

**Published:** 2012-03-06

**Authors:** Yunjun Yang, Jingfei Luan

**Affiliations:** State Key Laboratory of Pollution Control and Resource Reuse, School of the Environment, Nanjing University, Nanjing 210093, China; Email: yyj@boyone.net

**Keywords:** polyaniline/Bi_2_SnTiO_7_, photocatalytic activity, methylene blue, visible light irradiation, photodegradation pathway

## Abstract

A novel polyaniline/Bi_2_SnTiO_7_ composite polymer was synthesized by chemical oxidation *in-situ* polymerization method and sol-gel method for the first time. The structural properties of novel polyaniline/Bi_2_SnTiO_7_ have been characterized by X-ray diffraction, scanning electron microscopy, X-ray photoelectron spectroscopy and X-ray spectrometry. The lattice parameter of Bi_2_SnTiO_7_ was found to be a = 10.52582(8) Å. The photocatalytic degradation of methylene blue was realized under visible light irradiation with the novel polyaniline/Bi_2_SnTiO_7_ as catalyst. The results showed that novel polyaniline/Bi_2_SnTiO_7_ possessed higher catalytic activity compared with Bi_2_InTaO_7_ or pure TiO_2_ or N-doped TiO_2_ for photocatalytic degradation of methylene blue under visible light irradiation. The photocatalytic degradation of methylene blue with the novel polyaniline/Bi_2_SnTiO_7_ or N-doped TiO_2_ as catalyst followed first-order reaction kinetics, and the first-order rate constant was 0.01504 or 0.00333 min^−1^. After visible light irradiation for 220 minutes with novel polyaniline/Bi_2_SnTiO_7_ as catalyst, complete removal and mineralization of methylene blue was observed. The reduction of the total organic carbon, the formation of inorganic products, SO_4_^2−^ and NO_3−_, and the evolution of CO_2_ revealed the continuous mineralization of methylene blue during the photocatalytic process. The possible photocatalytic degradation pathway of methylene blue was obtained under visible light irradiation.

## 1. Introduction

Dye effluents from textile industries are becoming a serious environmental problem because of their toxicity, high chemical oxygen demand content, and biological degradation [1]. A lot of conventional methods have been proposed to treat industrial effluents, but each method has its shortcomings [1,2,3,4,5,6,7]. In the last decade, photocatalytic degradation processes have been widely applied as techniques for the destruction of organic pollutants in wastewater and effluents, especially degradation of dyes [1,7,8,9,10,11,12,13,14,15,16,17,18,19,20,21]. Among various dyes, methylene blue (MB) dye is difficult to degrade and is often utilized as a model dye contaminant to evaluate the activity of a photocatalyst both under ultraviolet light irradiation [18,19,22] and under visible light irradiation [20,21,22,23,24]. There are many reports on the photodegradation of MB. Unfortunately, most of these experiments were carried out under ultraviolet light irradiation. Up to now, there were only a few reports for MB dye degradation under visible light irradiation such as the research of Asahi *et al.* with a reduced TiO_x_ (TiO_2−x_N_x_) as catalyst and the research of Li *et al.* with Pt–TiO_2_ as photocatalyst [21,24]. Zhang [25] utilized N-doped TiO_2_ as catalyst to degrade MB under visible light irradiation and found that the removal ratio of MB was only 35% after visible light irradiation for 180 minutes. It is known that ultraviolet light only occupies 4% of the solar energy spectrum. For this reason, there is great interest in developing new visible light-responsive photocatalysts capable of utilizing the more ample visible light spectrum, which occupies about 43% of the solar energy range. Therefore, there is an urgent need to develop novel visible light-responsive photocatalysts. 

With the development of investigations into photocatalysis processes, investigators have also paid considerable attention to researching and developing novel photocatalysts [26,27,28,29]. Moreover, UV-diffuse reflectance spectroscopy and the bandgap of novel photocatalysts [30,31] also play an important role in any photocatalyst system. Up until recently, TiO_2_ was the most common photocatalyst, but TiO_2_ could not be utilized in the visible light region and could only degrade MB under ultraviolet light irradiation. Therefore, some efficient catalysts which could generate electron-hole pairs under visible light irradiation should be developed. Fortunately, A_2_B_2_O_7_ compounds were often considered to have photocatalytic properties under visible light irradiation. In our previous work [32], we had found that Bi_2_InTaO_7_ crystallized with the pyrochlore-type structure and acted as a photocatalyst under visible light irradiation and seemed to have potential for improvement of photocatalytic activity by modification of its structure. According to above analysis, we could assume that substitution of Ta^5+^ and In^3+^ by Sn^4+^ and Ti^4+^ in Bi_2_InTaO_7_ might increase the carrier concentration. As a result, a change and improvement of the electron transport and photophysical properties could be found in the novel Bi_2_SnTiO_7_ compound which might display advanced photocatalytic properties. Moreover, owing to the excellent environmental stability of polyaniline, the polyaniline-hybridized Bi_2_SnTiO_7_ sample should possess more advanced photocatalytic properties. 

Bi_2_SnTiO_7_ had never been produced and the data about its structural and photophysical properties such as space group and lattice constants had not been previously reported. In addition, the photocatalytic properties of Bi_2_SnTiO_7_ had not been investigated by other investigators. The molecular composition of Bi_2_SnTiO_7_ was very similar to that of other A_2_B_2_O_7_ compounds. Thus the resemblance suggested that Bi_2_SnTiO_7_ and the polyaniline-hybridized Bi_2_SnTiO_7_ might possess photocatalytic properties under visible light irradiation, like other members in the A_2_B_2_O_7_ family. Bi_2_SnTiO_7_ also seemed to have potential for improvement of photocatalytic activity by modification of its structure because it had been proved that a slight modification of a semiconductor structure would cause a remarkable change in photocatalytic properties [21]. In this paper, Bi_2_SnTiO_7_ was prepared for the first time by the solid-state reaction method and the novel composite polymer polyaniline/Bi_2_SnTiO_7_ was synthesized by the chemical oxidation *in-situ* polymerization method and sol-gel method for the first time. The structural and photocatalytic properties of the polyaniline-hybridized Bi_2_SnTiO_7_ were investigated in detail. The photocatalytic degradation of MB under visible light irradiation was also performed to evaluate the photocatalytic activity of the polyaniline-hybridized Bi_2_SnTiO_7_. A comparison among the photocatalytic properties of the polyaniline-hybridized Bi_2_SnTiO_7_, Bi_2_InTaO_7_ and N-doped TiO_2_ was carried out in order to elucidate the structure-photocatalytic activity relationship in the polyaniline-hybridized Bi_2_SnTiO_7_.

## 2. Results and Discussion

### 2.1. Crystal Structure of Bi_2_SnTiO_7_

**Figure 1:**
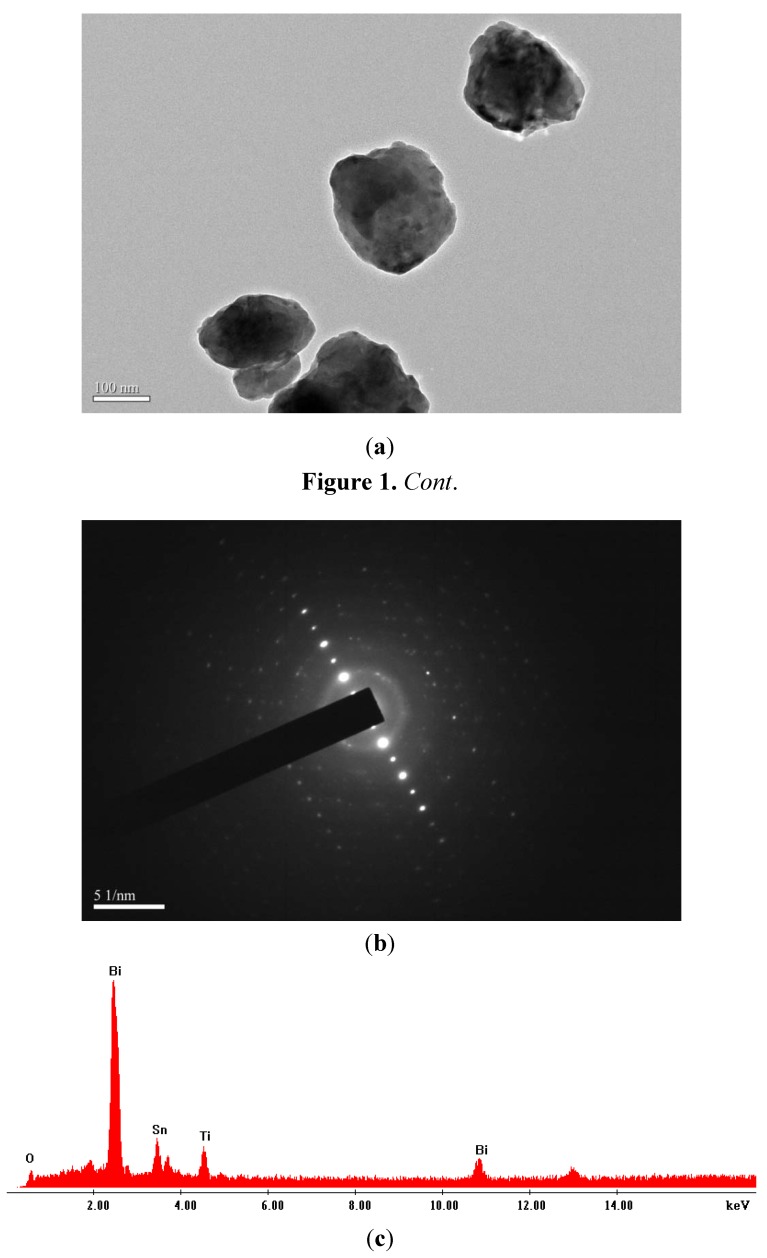
TEM image of Bi_2_SnTiO_7_ (**a**) and the selected area electron diffraction pattern of Bi_2_SnTiO_7_ (**b**) and SEM-EDS spectrum of Bi_2_SnTiO_7_ (**c**).

Figure 1 presents a TEM image, the selected area electron diffraction pattern and the SEM-EDS spectrum of Bi_2_SnTiO_7_. The TEM image of Bi_2_SnTiO_7_ showed that the morphology of the Bi_2_SnTiO_7_ particle was roundish and the Bi_2_SnTiO_7_ particle size was uniform. It could be seen that the Bi_2_SnTiO_7_ particles crystallized well and the average particle size of Bi_2_SnTiO_7_ was about 180 nm. The SEM-EDS spectrum of Bi_2_SnTiO_7_ revealed that Bi_2_SnTiO_7_ was pure phase without any other impurities and Bi_2_SnTiO_7_ displayed the presence of bismuth, tin, titanium and oxygen. It could be seen from Figure 1 that Bi_2_SnTiO_7_ crystallized with the pyrochlore-type structure, cubic crystal system and space group *Fd3m*.

The lattice parameter for Bi_2_SnTiO_7_ was proved to be a = 10.52582(8) Å. According to the calculation results from Figure 1, the (h k l) value for the main peaks of Bi_2_SnTiO_7_ could be found and indexed.

**Figure 2:**
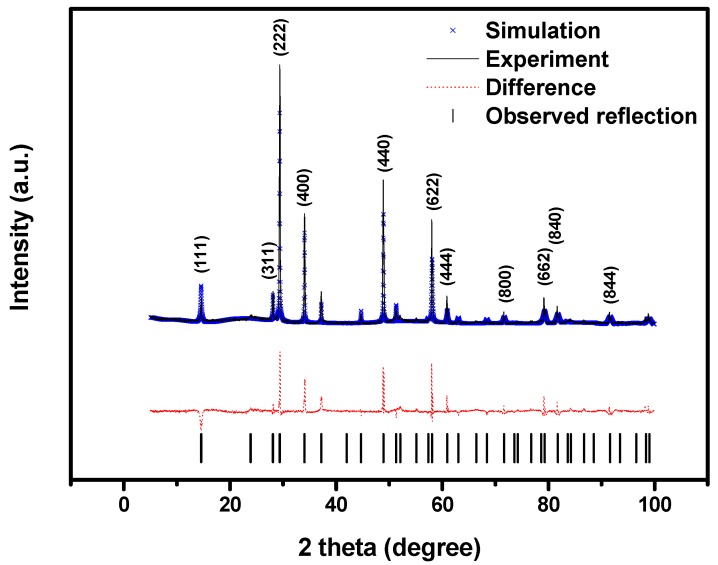
X-ray powder diffraction pattern and Rietveld refinements of Bi_2_SnTiO_7_ prepared by a solid-state reaction method at 1,100 °C.

Full-profile structure refinements of the collected X-ray diffraction data of Bi_2_SnTiO_7_ were obtained by the RIETAN^TM^ [33] program, which was based on Pawley analysis. The refinement results of Bi_2_SnTiO_7_ are shown in Figure 2. The atomic coordinates and structural parameters of Bi_2_SnTiO_7_ are listed in Table 1. The results of the final refinement for Bi_2_SnTiO_7_ indicated a good agreement between the observed and calculated intensities in a pyrochlore-type structure and cubic crystal system with space group *Fd3m*. Our XRD results also showed that Bi_2_SnTiO_7_ and Bi_2_InTaO_7_ crystallized in the same structure, and 2 theta angles of each reflection of Bi_2_SnTiO_7_ changed with Sn^4+^ and Ti^4+^ being replaced by In^3+^ and Ta^5+^. Bi_2_InTaO_7_ also crystallized with a cubic structure by space group Fd3m and the lattice parameter of Bi_2_InTaO_7_ was a = 10.74641(0) Å. The lattice parameter of Bi_2_SnTiO_7_ was a = 10.52582(8) Å, which indicated that the lattice parameter of Bi_2_SnTiO_7_ decreased compared with the lattice parameter of Bi_2_InTaO_7_ because the In^3+^ ionic radii (0.92 Å) was larger than the Sn^4+^ ionic radii (0.71Å) and the Ta^5+^ ionic radii (0.68 Å) was equal to the Ti^4+^ ionic radii (0.68Å). The outcome of refinement for Bi_2_SnTiO_7_ generated the unweighted *R* factor, *R*_P_ = 10.72% with space group *Fd3m*. Zou *et al.* [34] refined the crystal structure of Bi_2_InNbO_7_ and obtained a large *R* factor for Bi_2_InNbO_7_, which was ascribed to a slightly modified structure model for Bi_2_InNbO_7_. According to the high purity of the precursors which were utilized in this study and the fact which the EDS results did not trace any other elements, it was unlikely that the observed space groups originated from the presence of impurities. Therefore, it was suggested that the slightly high *R* factor for Bi_2_SnTiO_7_ was owing to a slightly modified structure model for Bi_2_SnTiO_7_. It should be emphasized that the defects or the disorder/order of a fraction of the atoms could cause the change of structures, including different bond-distance distributions, thermal displacement parameters and/or occupation factors for some of the atoms. 

**Table 1:** Atomic coordinates and structural parameters of Bi_2_SnTiO_7_ prepared by the solid state reaction method.

Atom	x	y	z	Occupation factor
Bi	0.0000	0	0	1.0
Sn	0.5000	0.5000	0.5000	0.5
Ti	0.5000	0.5000	0.5000	0.5
O(1)	−0.0947	0.1250	0.1250	1.0
O(2)	0.1250	0.1250	0.1250	1.0

In order to reveal the surface chemical compositions and the valence states of various elements of Bi_2_SnTiO_7_, the X-ray photoelectron spectrum of Bi_2_SnTiO_7_ for detecting Bi, Sn, Ti and O was performed. The full XPS spectrum confirmed that the prepared Bi_2_SnTiO_7_ contained the elements Bi, Sn, Ti and O, which was consistent with the SEM-EDS results. The different elemental peaks for Bi_2_SnTiO_7_ which are corresponding to definite bind energies are given in Table 2. The results illustrated that the oxidation states of Bi, Sn, Ti and O ions from Bi_2_SnTiO_7_ were +3, +4, +4 and −2, respectively. Moreover, the average atomic ratio of Bi:Sn:Ti:O for Bi_2_SnTiO_7_ was 2.00:0.98:1.02:6.97 accoring to our XPS, SEM-EDS and XFS results. Accordingly, it could be deduced that the resulting material was highly pure under our preparation conditions. It was remarkable that there were not any shoulders and widening in the XPS peaks of Bi_2_SnTiO_7_, which suggested the absence of any other phases. 

**Table 2:** Binding energies (BE) for key elements from Bi_2_SnTiO_7_.

Compound	Bi_4f__7/2_	Sn_3d5/2_	Ti_3p_	O_1s_
	BE (eV)	BE (eV)	BE (eV)	BE (eV)
Bi_2_SnTiO_7_	159.50	486.55	37.50	530.15

### 2.2. Photocatalytic Properties

**Figure 3:**
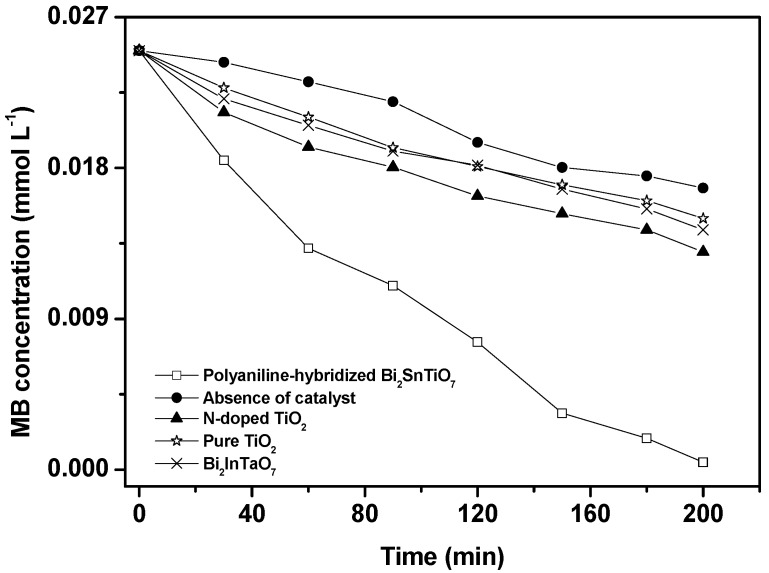
Photocatalytic degradation of methylene blue under visible light irradiation in the presence of the polyaniline-hybridized Bi_2_SnTiO_7_, Bi_2_InTaO_7_, pure TiO_2_, N-doped TiO_2_ as well as in the absence of a photocatalyst.

Generally, the direct absorption of band-gap photons would result in the generation of electron–hole pairs within the polyaniline-hybridized Bi_2_SnTiO_7_, subsequently, the charge carriers began to diffuse to the surface of the polyaniline-hybridized Bi_2_SnTiO_7_. As a result, the photocatalytic activity for decomposing organic compounds with the polyaniline-hybridized Bi_2_SnTiO_7_ might be enhanced. Changes in the UV-Vis spectrum of MB upon exposure to visible light (*λ* > 400 nm) irradiation with the presence of the polyaniline-hybridized Bi_2_SnTiO_7_, Bi_2_InTaO_7_ or N-doped TiO_2_ indicated that the polyaniline-hybridized Bi_2_SnTiO_7_, Bi_2_InTaO_7_ or N-doped TiO_2_ could photodegrade MB effectively under visible light irradiation. Figure 3 shows the photocatalytic degradation of methylene blue under visible light irradiation in the presence of the polyaniline-hybridized Bi_2_SnTiO_7_, Bi_2_InTaO_7_, pure TiO_2_, N-doped TiO_2_ as well as in the absence of a photocatalyst. 

The results showed that a reduction in the typical MB peaks at 665 nm and 614 nm was clearly noticed and the photodegradation rate of MB was about 2.046 × 10^−9^ mol L^−1^ s^−1^ and the photonic efficiency was estimated to be 0.0430% (*λ* = 420 nm) with the polyaniline-hybridized Bi_2_SnTiO_7_ as catalyst. Similarly, the photodegradation rate of MB was about 1.001 × 10^−9^ mol L^−1^ s^−1^ and the photonic efficiency was estimated to be 0.0210% (*λ* = 420 nm) with N-doped TiO_2_ as catalyst. Furthermore, the photodegradation rate of MB was about 0.891 × 10^−9^ mol L^−1^ s^−1^ and the photonic efficiency was estimated to be 0.0187% (*λ* = 420 nm) with Bi_2_InTaO_7_ as catalyst. As a contradistinction, the photodegradation rate of MB within 200 minutes of visible light irradiation was only 0.8338 × 10^−9^ mol L^−1^ s^−1^ and the photonic efficiency was estimated to be 0.0175% (*λ* = 420 nm) with pure TiO_2_ as catalyst. The photodegradation rate of MB was about 0.6830 × 10^−9^ mol L^−1^ s^−1^ and the photonic efficiency was estimated to be 0.0143% (*λ* = 420 nm) in the absence of a photocatalyst. The results showed that the photodegradation rate of MB and the photonic efficiency with the polyaniline-hybridized Bi_2_SnTiO_7_ as catalyst were both higher than those with N-doped TiO_2_ or Bi_2_InTaO_7_ or pure TiO_2_ as catalyst. The photodegradation rate of MB and the photonic efficiency with N-doped TiO_2_ as catalyst were both higher than those with Bi_2_InTaO_7_ or pure TiO_2_ as catalyst. The photodegradation rate of MB and the photonic efficiency with Bi_2_InTaO_7_ as catalyst were both higher than those with pure TiO_2_ or the absence of a photocatalyst. The photodegradation rate of MB and the photonic efficiency with pure TiO_2_ as catalyst were both higher than those with the absence of a photocatalyst. When the polyaniline-hybridized Bi_2_SnTiO_7_, N-doped TiO_2_, Bi_2_InTaO_7_ or pure TiO_2_ was utilized as catalyst, the photodegradation conversion rate of MB was 98.20%, 48.05%, 42.76% and 40.02% after visible light irradiation for 200 minutes, respectively. Furthermore, the photodegradation conversion rate of MB was 32.78% after visible light irradiation for 200 minutes with the absence of a photocatalyst because of the MB dye photo-sensitization effect [35]. After visible light irradiation for 220 minutes with the polyaniline-hybridized Bi_2_SnTiO_7_ as catalyst, complete removal of MB was observed and the complete disappearance of the absorption peaks which presented the absolute color change from deep blue into colorless solution occurred. 

Bi_2_SbVO_7_, Fe_2_BiSbO_7_ and SrFeO_3_ were also prepared by using the preparation methods from the literature reports [36,37,38]. Polyaniline-hybridized Bi_2_SnTiO_7_, Bi_2_SbVO_7_, Fe_2_BiSbO_7_ and SrFeO_3_ were utilized as catalysts to degrade MB under the same experimental conditions which were shown in our paper. The results are shown in Table 3. It could be seen from Table 3 that the removal rate of MB was 98.2%, 96.5%, 95.0% or 59.7% with polyaniline-hybridized Bi_2_SnTiO_7_, Bi_2_SbVO_7_, Fe_2_BiSbO_7_ or SrFeO_3_ as catalyst within 200 minutes under visible light irradiation. The results showed that the polyaniline-hybridized Bi_2_SnTiO_7_ showed the best photocatalytic activity for photodegradation of MB compared with Bi_2_SbVO_7_, Fe_2_BiSbO_7_ or SrFeO_3_. Furthermore, our N-doped TiO_2_ showed low photocatalytic activity for photodegradation of MB compared with the N-doped TiO_2_ which was produced by Yang *et al.* [39] under visible light irradiation. The reason was that N:TiO_2_ molar ratio was different between our N-doped TiO_2_ and Yang’s N-doped TiO_2_ [39], which indicated that our N-doped TiO_2_ contained 62% anatase phase and 38% rutile phase, at the same time, Yang’s N-doped TiO_2_ [39] contained 100% anatase phase. We prepared N-doped TiO_2_ by using the preparation method from Yang *et al.* and we found that N-doped TiO_2_ showed similar results for degradation of MB.

**Table 3:** The removal rate of MB by using different catalysts within 200 minutes under visible light irradiation.

Catalyst	Polyaniline-hybridized Bi_2_SnTiO_7_	Bi_2_SbVO_7_	Fe_2_BiSbO_7_	SrFeO_3_
Removal rate of MB (%)	98.2	96.5	95.0	59.7

According to above results, the photocatalytic degradation activity of the polyaniline-hybridized Bi_2_SnTiO_7_ was much higher than that of N-doped TiO_2_, Bi_2_InTaO_7_ or pure TiO_2_. Meanwhile, N-doped TiO_2_ showed higher photocatalytic degradation activity for MB photodegradation compared with Bi_2_InTaO_7_ or pure TiO_2_. Bi_2_InTaO_7_ showed higher photocatalytic degradation activity for MB photodegradation compared with pure TiO_2_. Pure TiO_2_ was more suitable for MB photodegradation than the absence of a photocatalyst. The photocatalytic property of novel polyaniline-hybridized Bi_2_SnTiO_7_ under visible light irradiation was amazing compared with that of N-doped TiO_2_ or pure TiO_2_, and the main reason was that the specific surface area of the polyaniline-hybridized Bi_2_SnTiO_7_ was much smaller than that of N-doped TiO_2_ or pure TiO_2_. BET isotherm measurements of the polyaniline-hybridized Bi_2_SnTiO_7_, N-doped TiO_2_ and pure TiO_2_ provided a specific surface area of 4.29 m^2^ g^−1^, 45.53 m^2^ g^−1^ and 46.24 m^2^ g^−1^ respectively, which indicated that the photocatalytic degradation activity of the polyaniline-hybridized Bi_2_SnTiO_7_ could be improved consumedly by enhancing the specific surface area of the polyaniline-hybridized Bi_2_SnTiO_7_. 

**Figure 4:**
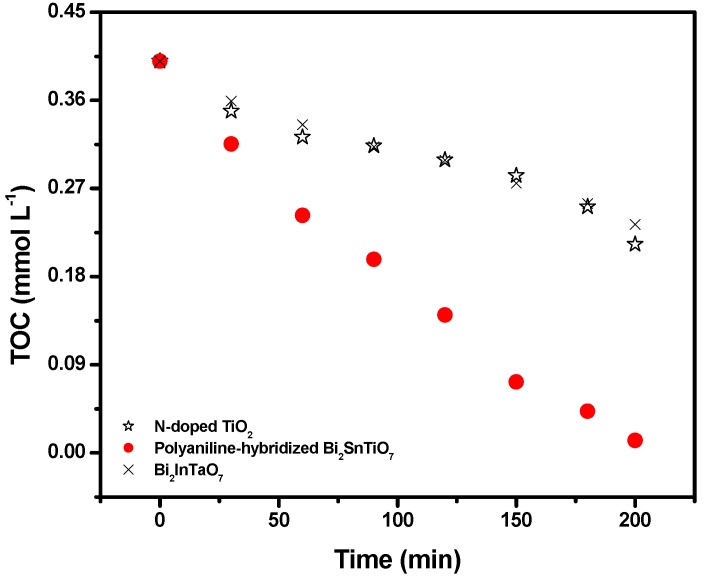
Disappearance of total organic carbon (TOC) during photocatalytic degradation of methylene blue with the polyaniline-hybridized Bi_2_SnTiO_7_, Bi_2_InTaO_7_ or N-doped TiO_2_ as catalyst under visible light irradiation.

Figure 4 shows the change of TOC during photocatalytic degradation of MB with the polyaniline-hybridized Bi_2_SnTiO_7_, Bi_2_InTaO_7_ or N-doped TiO_2_ as catalyst under visible light irradiation. 

The TOC measurements revealed the disappearance of organic carbon when the MB solution which contained the polyaniline-hybridized Bi_2_SnTiO_7_, Bi_2_InTaO_7_ or N-doped TiO_2_ was exposed under visible light irradiation. The results showed that 96.77% or 46.77% or 41.71% of TOC decrease was obtained after visible light irradiation for 200 minutes when the polyaniline-hybridized Bi_2_SnTiO_7_ or N-doped TiO_2_ or Bi_2_InTaO_7_ was utilized as photocatalyst. Consequently, after visible light irradiation for 220 minutes with the polyaniline-hybridized Bi_2_SnTiO_7_ as catalyst, the entire mineralization of MB was observed because of 100% TOC removal. The turnover number which represented the ratio between the total amount of evolved gas and dissipative catalyst was calculated to be more than 0.258 for the polyaniline-hybridized Bi_2_SnTiO_7_ after 200 minutes of reaction time under visible light irradiation and this turnover number was evident to prove that this reaction occurred catalytically. Similarly, when the light was turned off in this experiment, the stop of this reaction showed the obvious light response.

**Figure 5:**
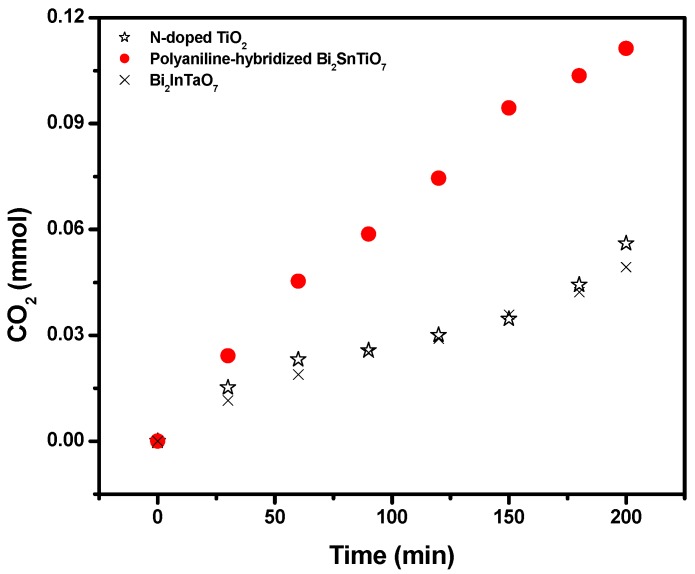
CO_2_ production kinetics during the photocatalytic degradation of methylene blue with the polyaniline-hybridized Bi_2_SnTiO_7_, Bi_2_InTaO_7_ or N-doped TiO_2_ as catalyst under visible light irradiation.

Figure 5 shows the amount of CO_2_ which was released during the photodegradation of MB with the polyaniline-hybridized Bi_2_SnTiO_7_, Bi_2_InTaO_7_ or N-doped TiO_2_ as catalyst under visible light irradiation. The amount of CO_2_ increased gradually with increasing reaction time when MB was photodegraded by the polyaniline-hybridized Bi_2_SnTiO_7_, Bi_2_InTaO_7_ or N-doped TiO_2_. At the same time, after visible light irradiation of 200 minutes, the CO_2_ production of 0.11129 mmol with the polyaniline-hybridized Bi_2_SnTiO_7_ as catalyst was higher than the CO_2_ production of 0.05600 mmol with N-doped TiO_2_ as catalyst. Meanwhile after visible light irradiation of 200 minutes, the CO_2_ production of 0.05600 mmol with N-doped TiO_2_ as catalyst was higher than the CO_2_ production of 0.04934 mmol with Bi_2_InTaO_7_ as catalyst.

**Figure 6:**
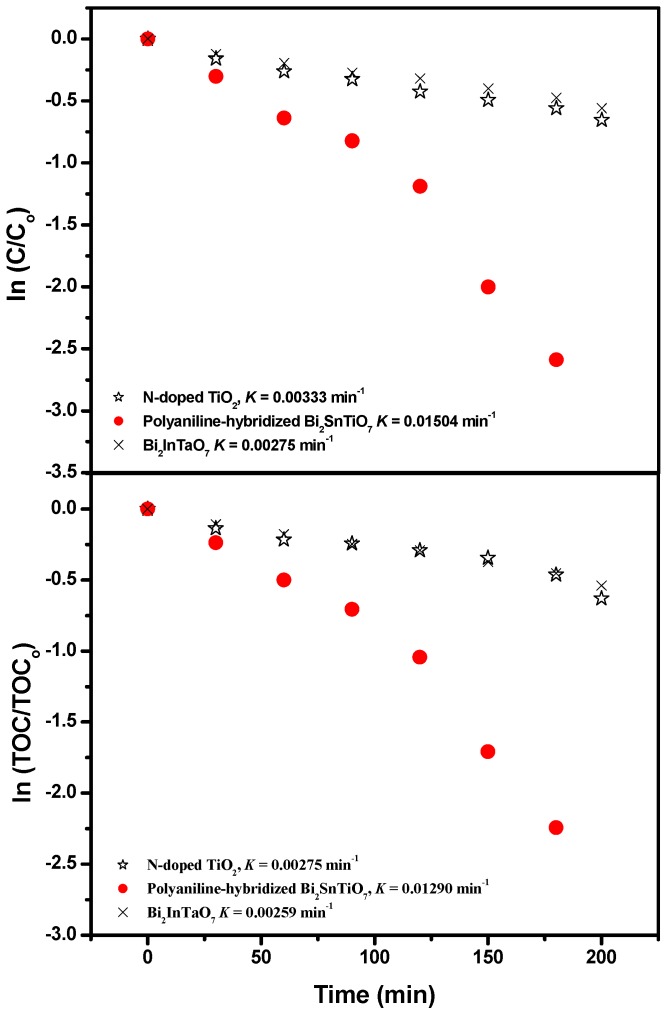
Observed first-order kinetic plots for the photocatalytic degradation of methylene blue with the polyaniline-hybridized Bi_2_SnTiO_7_, Bi_2_InTaO_7_ or N-doped TiO_2_ as catalyst under visible light irradiation.

The first-order nature of the photocatalytic degradation kinetics with the polyaniline-hybridized Bi_2_SnTiO_7_ or Bi_2_InTaO_7_ or N-doped TiO_2_ as catalyst is clearly demonstrated in Figure 6. The results showed a linear correlation between ln (*C/C_o_*) (or ln (*TOC/TOC_o_*)) and the irradiation time for the photocatalytic degradation of MB under visible light irradiation with the presence of the polyaniline-hybridized Bi_2_SnTiO_7_ or Bi_2_InTaO_7_ or N-doped TiO_2_. Here, *C* represented the MB concentration at time t, and *C_o_* represented the initial MB concentration, and *TOC* represented the total organic carbon concentration at time t, and *TOC_o_* represented the initial total organic carbon concentration. According to Figure 6, the first-order rate constant *k_C_* of MB concentration was estimated to be 0.01504 min^−1^ with the polyaniline-hybridized Bi_2_SnTiO_7_ as catalyst, 0.00275 min^−1^ with Bi_2_InTaO_7_ as catalyst and 0.00333 min^−1^ with N-doped TiO_2_ as catalyst. The different value of *k_C_* indicated that the polyaniline-hybridized Bi_2_SnTiO_7_ was more suitable for the photocatalytic degradation of MB under visible light irradiation than N-doped TiO_2_ or Bi_2_InTaO_7_. Meanwhile N-doped TiO_2_ was more suitable for the photocatalytic degradation of MB under visible light irradiation than Bi_2_InTaO_7_. Figure 6 also showed that the first-order rate constant *K_TOC_* of TOC was estimated to be 0.01290 min^−1^ with the polyaniline-hybridized Bi_2_SnTiO_7_ as catalyst, 0.00275 min^−1^ with N-doped TiO_2_ as catalyst and 0.00259 min^−1^ with Bi_2_InTaO_7_ as catalyst, which indicated that the photodegradation intermediate products of MB probably appeared during the photocatalytic degradation of MB under visible light irradiation because of the different value between *k_C_* and *K_TOC_*. It could also be seen from Figure 6 that the polyaniline-hybridized Bi_2_SnTiO_7_ showed higher mineralization efficiency for MB degradation compared with N-doped TiO_2_ or Bi_2_InTaO_7_. At the same time, N-doped TiO_2_ showed higher mineralization efficiency for MB degradation compared with Bi_2_InTaO_7_.

**Figure 7:**
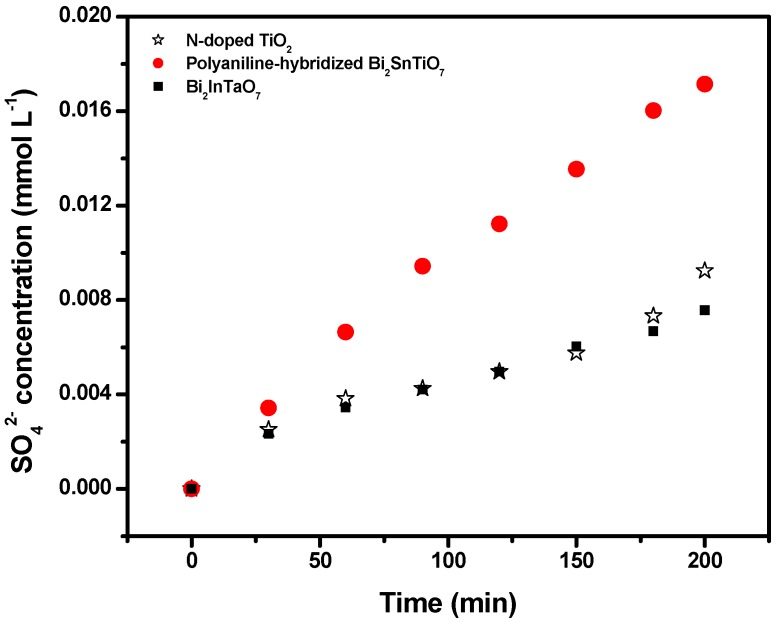
The concentration variation of SO_4_^2−^ during photocatalytic degradation of methylene blue with the polyaniline-hybridized Bi_2_SnTiO_7_, Bi_2_InTaO_7_ or N-doped TiO_2_ as catalyst under visible light irradiation.

**Figure 8:**
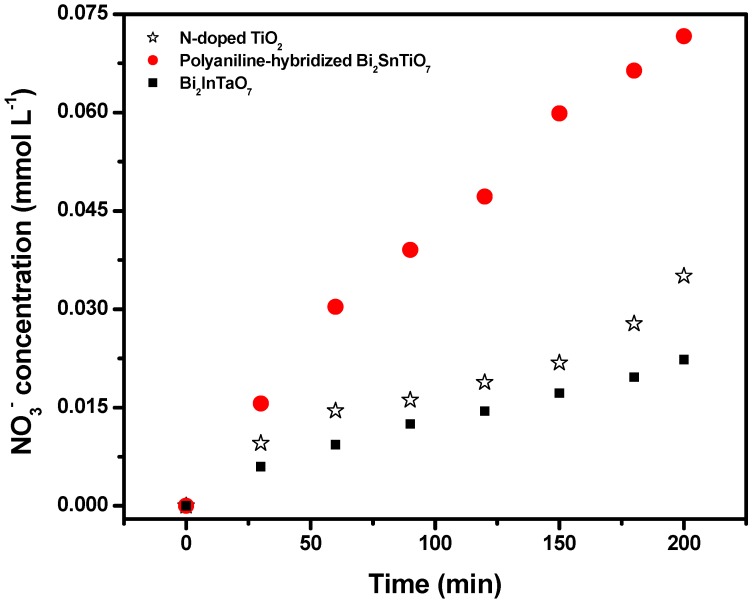
The concentration variation of NO_3_^−^ during photocatalytic degradation of methylene blue with the polyaniline-hybridized Bi_2_SnTiO_7_, Bi_2_InTaO_7_ or N-doped TiO_2_ as catalyst under visible light irradiation.

Some inorganic ions such as NH_4_^+^, NO_3_^−^ and SO_4_^2−^ were formed in parallel as the end products of nitrogen and sulfur atoms which existed in MB. Figure 7 and Figure 8 show the concentration variation of SO_4_^2−^ and NO_3−_ during photocatalytic degradation of MB with the polyaniline-hybridized Bi_2_SnTiO_7_ or Bi_2_InTaO_7_ or N-doped TiO_2_ as catalyst under visible light irradiation. The results showed that the concentration of NO_3−_ or SO_4_^2−^ increased gradually with increasing reaction time when MB was photodegraded by the polyaniline-hybridized Bi_2_SnTiO_7_ or Bi_2_InTaO_7_ or N-doped TiO_2_. Monitoring the presence of ions in the solution revealed that the SO_4_^2–^ ion concentration was 0.01714 mM or 0.00924 mM or 0.00757 mM with the polyaniline-hybridized Bi_2_SnTiO_7_ or N-doped TiO_2_ or Bi_2_InTaO_7_ as catalyst after visible light irradiation for 200 minutes, indicating that 68.56% or 36.94% or 30.28% of sulfur from MB was converted into sulfate ions with the polyaniline-hybridized Bi_2_SnTiO_7_ or N-doped TiO_2_ or Bi_2_InTaO_7_ as catalyst after visible light irradiation for 200 minutes. 

It could be seen from Figure 8 that the NO_3_^−^ ion concentration was 0.07165 mM or 0.0351 mM or 0.02232 mM with the polyaniline-hybridized Bi_2_SnTiO_7_ or N-doped TiO_2_ or Bi_2_InTaO_7_ as catalyst after visible light irradiation for 200 minutes, indicating that 95.53% or 46.80% or 29.76% of nitrogen from MB was converted into nitrate ions with the polyaniline-hybridized Bi_2_SnTiO_7_ or N-doped TiO_2_ or Bi_2_InTaO_7_ as catalyst after visible light irradiation for 200 minutes. The sulfur was first hydrolytically removed, and subsequently was oxidized and transformed into SO_4_^2−^. At the same time, nitrogen atoms in the −3 oxidation state prduced NH_4_^+^ cations that subsequently were oxidized into NO_3__−_ ions. As expected, the formation kinetics with the polyaniline-hybridized Bi_2_SnTiO_7_ was significantly faster than that of N-doped TiO_2_ or Bi_2_InTaO_7_ by using the same amount of photocatalyst. Moreover, the formation kinetics with N-doped TiO_2_ was faster than that of Bi_2_InTaO_7_ when using the same amount of photocatalyst. It was noteworthy that the amount of SO_4_^2−^ ions which was released into the solution was lower than the amount of SO_4_^2−^ which should come from stoichiometry. One possible reason could be a loss of sulfur-containing volatile compounds such as SO_2_. The second possible reason was a partially irreversible adsorption of some SO_4_^2−^ ions on the surface of the photocatalyst which had been observed by Lachheb *et al.* by titanium dioxide [40]. Regardless whether the sulfate ions were adsorbed irreversibly on the surface or not, it was important to stress that the evidence for restrained photocatalytic activity was not noticed. 

**Scheme 1:**
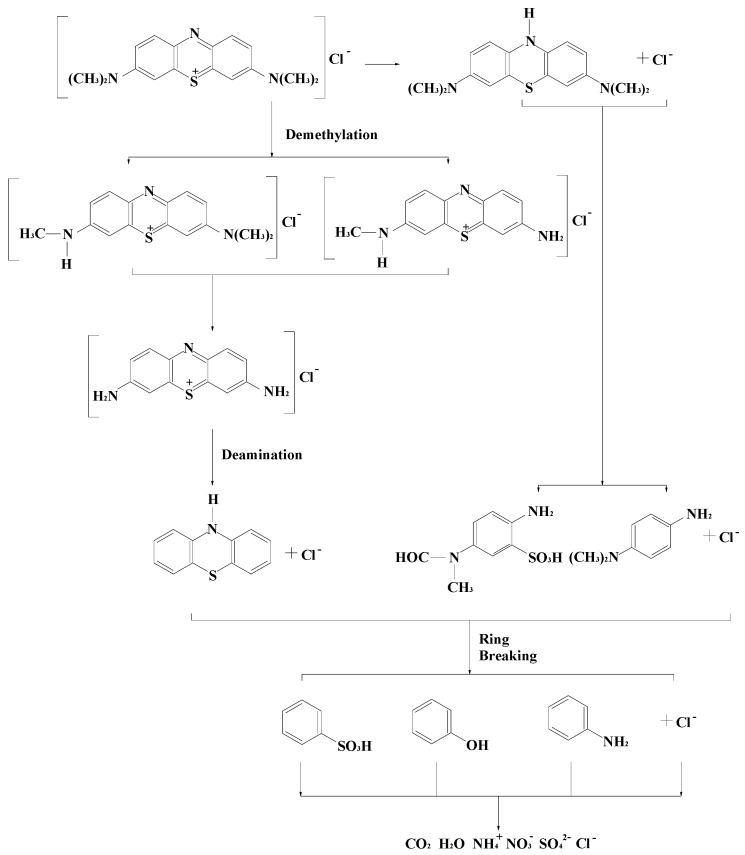
Suggested photocatalytic degradation pathway scheme for methylene blue under visible light irradiation in the presence of the polyaniline-hybridized Bi_2_SnTiO_7_.

The photodegradation intermediate products of MB (*m/z* = 284.1) in our experiment were identified as azure B (*m/z* = 270.1), azure C (*m/z* = 242.1), thionine (*m/z* = 228.0), leucomethylene blue (*m/z* = 285.0) and aniline (*m/z* = 93.0). According to the intermediate products which were found in this work and the observed appearance time of other intermediate products, a possible photocatalytic degradation pathway for MB was proposed. Scheme 1 shows the suggested photocatalytic degradation pathway scheme for methylene blue under visible light irradiation in the presence of the polyaniline-hybridized Bi_2_SnTiO_7_. The molecule of MB was converted to small organic species, which were subsequently mineralized into inorganic products such as SO_4_^2−^ ions, NO_3__−_ ions, CO_2_ and water ultimately.

### 2.3. Photocatalytic Degradation Mechanism

**Scheme 2:**
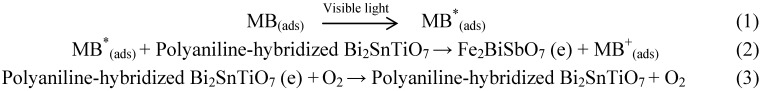
Photosensitization effect by the dye MB.

The action spectra of MB degradation with the polyaniline-hybridized Bi_2_SnTiO_7_ as catalyst was observed under visible light irradiation. A clear photonic efficiency (0.0112% at its maximal point) at wavelengths which corresponded to sub-Eg energies of the photocatalysts (*λ* from 490 nm to 800 nm) was observed for the polyaniline-hybridized Bi_2_SnTiO_7_. The existence of photonic efficiency at this region revealed that photons were not absorbed by the photocatalysts. In particular, the correlation between the low-energy action spectrum and the absorption spectrum of MB clearly demonstrated that any photodegradation results at wavelengths above 490 nm should be attributed to photosensitization effect by the dye MB itself (Scheme 2). 

According to the mechanism which was shown in Scheme 2, MB which was adsorbed on the polyaniline-hybridized Bi_2_SnTiO_7_ was excited by visible light irradiation. Subsequently, an electron was injected from the excited MB to the conduction band of the polyaniline-hybridized Bi_2_SnTiO_7_ where the electron was scavenged by molecular oxygen. Scheme I explained the results which were obtained with the polyaniline-hybridized Bi_2_SnTiO_7_ as catalyst under visible light irradiation, where the polyaniline-hybridized Bi_2_SnTiO_7_ could serve to reduce recombination of photogenerated electrons and holes by scavenging of electrons [41]. 

**Scheme 3:**
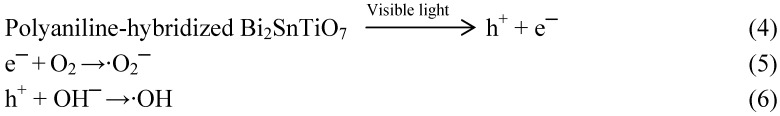
The observed mechanism for the polyaniline-hybridized Bi_2_SnTiO_7_ under supra-bandgap irradiation.

Below 490 nm, the situation was different. The results of photonic efficiency correlated well with the absorption spectra of the polyaniline-hybridized Bi_2_SnTiO_7_. These results evidently showed that the mechanism which was responsible for the photodegradation of MB went through band gap excitation of the polyaniline-hybridized Bi_2_SnTiO_7_. Despite the detailed experiments about the effect of oxygen and water were not performed, it was logical to presume that the mechanism in the first step was similar to the observed mechanism for the polyaniline-hybridized Bi_2_SnTiO_7_ under supra-bandgap irradiation, namely Scheme 3:

According to first principles calculations, we deduced that the conduction band of the polyaniline-hybridized Bi_2_SnTiO_7_ was composed of Ti 3*d* and Sn 5*p* orbital component, and the valence band of the polyaniline-hybridized Bi_2_SnTiO_7_ was composed of O 2*p* and Bi 6*s* orbital component. The polyaniline-hybridized Bi_2_SnTiO_7_ could produce electron–hole pairs by absorption of photons directly, and it indicated that enough energy which was larger than the band gap energy of the polyaniline-hybridized Bi_2_SnTiO_7_ was necessary for the photocatalytic degradation process of MB. 

Former luminescent studies had shown that the closer the M–O–M bond angle was to 180°, the more delocalized was the excited state [42]. As a result, the charge carriers could move more easily in the matrix. The mobility of the photoinduced electrons and holes influenced the photocatalytic activity because high diffusivity indicated the enhancement of probability that the photogenerated electrons and holes would reach the reactive sites of the catalyst surface. According to above results, the lattice parameter a = 10.52582(8) Å for Bi_2_SnTiO_7_ was smaller than the lattice parameter α = 10.70352(7) Å for Bi_2_InTaO_7_, thus the photoinduced electrons and holes inside Bi_2_SnTiO_7_ was easier and faster to reach the reactive sites on the catalyst surface compared with those of Bi_2_InTaO_7_, which showed the photocatalytic degradation activity of Bi_2_SnTiO_7_ was higher than that of Bi_2_InTaO_7_. For the polyaniline-hybridized Bi_2_SnTiO_7_ in this experiment, the Sn–O–Ti bond angle was 140.196° and the In–O–Ta bond angle was 118.764°, indicating that the Sn–O–Ti or In–O–Ta bond angle was close to 180°. Thus the photocatalytic activity of the polyaniline-hybridized Bi_2_SnTiO_7_ was correspondingly higher. The crystal structures of Bi_2_SnTiO_7_ and Bi_2_InTaO_7_ were the same, but their electronic structures were considered to be somewhat different. For Bi_2_SnTiO_7_, Ti was 3*d*-block metal element, and Bi was 6*p*-block rare earth metal element, and Sn was 5*p*-block metal element, but for Bi_2_InTaO_7_, Ta was 5*d*-block metal element, and In was 5*p*-block metal element, indicating that the crystal structure and the electronic structure of the photocatalysts affect the photocatalytic activity. Based on above analysis, the difference of photocatalytic MB degradation between Bi_2_SnTiO_7_ and Bi_2_InTaO_7_ can be attributed mainly to the difference in their crystalline structure and electronic structure. The crystal structure and the electronic structure of the polyaniline-hybridized Bi_2_SnTiO_7_ and N-doped TiO_2_ were totally different. For N-doped TiO_2_, Ti was 3*d*-block metal element, indicating that the different photodegradation effect of MB between the polyaniline-hybridized Bi_2_SnTiO_7_ and N-doped TiO_2_ could be attributed mainly to the difference of their crystalline structure and electronic structure. 

The present results indicated that the polyaniline-hybridized Bi_2_SnTiO_7_-visible light photocatalysis system might be regarded as a practical method for treatment of diluted colored waste water. This system could be utilized for decolorization, purification and detoxification of textile, printing and dyeing industries in the long-day countries. Meanwhile, this system did not need high pressure of oxygen, heating or any chemical reagents. Much decolorized and detoxified water were flowed from our new system for treatment, and the results showed that the polyaniline-hybridized Bi_2_SnTiO_7_-visible light photocatalysis system might provide a valuable treatment for purifying and reusing colored aqueous effluents.

## 3. Experimental

### 3.1. Synthesis of the Polyaniline-hybridized Bi_2_SnTiO_7_ and N-doped TiO_2_

Bi_2_SnTiO_7_ powder was first synthesized by the solid-state reaction method. TiO_2_, SnO_2_, Bi_2_O_3_, In_2_O_3_ and Ta_2_O_5_ with 99.99% purity purchased from Sinopharm Group Chemical Reagent Co. (Shanghai, China) were used as raw materials that were used without further purification. All powders were dried at 200 °C for 4 h before synthesis. In order to synthesize Bi_2_SnTiO_7_, the precursors were stoichiometrically mixed in a quartz mortar, subsequently pressed into small columns and put into an alumina crucible. Finally, calcination was carried out at 1,100 °C for 30 h in an electric furnace (KSL 1700X, Hefei Kejing Materials Technology CO., LTD, China). Similarly, Bi_2_InTaO_7_ was synthesized by calcination at 1,050 °C for 46 h. After sintering and grounding within a quartz mortar, ultrafine Bi_2_SnTiO_7_ powder was fabricated. 

A polyaniline-hybridized Bi_2_SnTiO_7_ sample was prepared as follows: an amount of distilled aniline was added to 150 mL of 1M HCl, and subsequently stirred for 30 minutes to ensure that the aniline was totally dissolved. Subsequently, a certain percentage of Bi_2_SnTiO_7_ was added into above solution, sonicated for 30 minutes to obtain a dispersed solution, and then stirred for 1 h. Thirdly, 0.5 g mL^−1^ ammonium thiosulphate (HCl) was added into the solution slowly, subsequently the mixture was stirred for 24 h. Finally, the suspension was filtered, and the precipitate was washed with alcohol and water for many times and dried at 60 °C to obtain polyaniline-hybridized-Bi_2_SnTiO_7_.

Nitrogen-doped titania (N-doped TiO_2_) catalyst with tetrabutyl titanate as a titanium precursor was prepared by using the sol–gel method at room temperature. Tetrabutyl titanate (17 mL) and absolute ethyl alcohol (40 mL) were mixed as solution a; subsequently solution a was added dropwise under vigorous stirring into solution b that contained absolute ethyl alcohol (40 mL), glacial acetic acid (10 mL) and double distilled water (5 mL) to form a transparent colloidal suspension c. Subsequently aqueous ammonia with N/Ti proportion of 8 mol% was added into the resulting transparent colloidal suspension under vigorous stirring and stirred for 1 h. Finally, the xerogel was formed after being aged for 2 days. The xerogel was ground into powder which was calcined at 500 °C for 2 h, subsequently above powder was ground in agate mortar and screened by shaker to obtain N-doped TiO_2_ powders. 

### 3.2. Characterization of the Polyaniline-hybridized Bi_2_SnTiO_7_

The crystalline phase of Bi_2_SnTiO_7_ was analyzed by X-ray diffractometer (D/MAX-RB, Rigaku Corporation, Japan) with Cu*K*α radiation (*λ* = 1.54056). The patterns were collected at 295 K with a step-scan procedure in the range of 2*θ* = 10–100°. The step interval was 0.02° and the time per step was 1.2 s. The accelerating voltage and applied current were 40 kV and 40 mA, respectively. The chemical composition of the compound was determined by scanning electron microscope-X-ray energy dispersion spectrum (SEM-EDS, LEO 1530VP, LEO Corporation, Germany), X-ray fluorescence spectrometer (XFS, ARL-9800, ARL Corporation, Switzerland) and X-ray photoelectron spectroscopy (XPS, ESCALABMK-2, VG Scientific Ltd., U.K.). The particle morphology of Bi_2_SnTiO_7_ was measured by transmission electron microscope (Tecnal F20 S-Twin, FEI Corporation, USA). The Bi^3+^ content, Sn^4+^ content, Ti^4+^ content and O^2−^ content of Bi_2_SnTiO_7_ and the valence state of elements were also analyzed by X-ray photoelectron spectroscopy (XPS). The chemical composition within the depth profile of Bi_2_SnTiO_7_ was examined by the argon ion denudation method when X-ray photoelectron spectroscopy was utilized. The surface areas of Bi_2_SnTiO_7_ and N-doped TiO_2_ were measured by the Brunauer-Emmett-Teller (BET) method (MS-21, Quantachrome Instruments Corporation, USA) with N_2_ adsorption at liquid nitrogen temperature. The particle sizes of the photocatalysts were measured by Malvern's mastersize-2000 particle size analyzer (Malvern Instruments Ltd, U.K.).

### 3.3. Photocatalytic Activity Tests

The photocatalytic activity of the polyaniline-hybridized Bi_2_SnTiO_7 _ was evaluated with methylene blue (C_16_H_18_ClN_3_S) (Tianjin Bodi Chemical Co., Ltd., China) as a model material. The photoreaction was carried out in a photochemical reaction apparatus (Nanjing Xujiang Machine Plant, China). The internal structure of the reaction apparatus is as following: the lamp is put into a quartz hydrazine which is a hollow structure and located in the middle of the reactor. The recirculating water through the reactor maintains a near constant reaction temperature (20 °C) and the solution was continuously stirred and aerated. Twelve holes which were utilized to put quartz tubes evenly distributed around the lamp and the distance between the lamp and each hole was equal. Under magnetic stirring, the photocatalyst within the MB solution was in the state of suspension. In this paper, the photocatalytic degradation of the MB solution was performed with 0.3 g polyaniline-hybridized Bi_2_SnTiO_7_ in 300 mL 0.025 mM MB aqueous solution in quartz tubes with 500 W Xenon lamp (400 nm < *λ* < 800 nm) as visible-light source. Prior to visible light irradiation, the suspensions which contained the catalyst and MB dye were magnetically stirred in the dark for 45 minutes to ensure establishment of an adsorption/desorption equilibrium among the polyaniline-hybridized Bi_2_SnTiO_7_, the MB dye and atmospheric oxygen. During visible light illumination, the suspension was stirred at 500 rpm and the initial pH value of the MB solution was 7.0 without pH adjustment in the reaction process. Above experiments were performed under oxygen-saturation conditions ([O_2_]_sat_ = 1.02 × 10^−3^ M). One of the quartz tubes was taken out from the photochemical reaction apparatus at various time intervals. The suspension was filtered through 0.22 µm membrane filters. The filtrate was subsequently analyzed by a Shimadzu UV-2450 UV-Visible spectrometer with the detecting wavelength at 665 nm. The experimental error was found to be within ±2.2%.

The incident photon flux *I_o_* measured by a radiometer (Model FZ-A, Photoelectric Instrument Factory Beijing Normal University, China) was determined to be 4.76 × 10^−6^ Einstein L^−1^ s^−1^ under visible light irradiation (wavelength range of 400–700 nm). The incident photon flux on the photoreactor was varied by adjusting the distance between the photoreactor and the Xe arc lamp. pH adjustment was not carried out and the initial pH value was 7.0. The inorganic products which were obtained from MB degradation were analyzed by ion chromatograph (DX-300, Dionex Corporation, USA). The identification of MB and the degradation intermediate products of MB were performed by liquid chromatograph―mass spectrometer (LC-MS, Thermo Quest LCQ Duo, USA, Beta Basic-C_18_ HPLC column: 150 × 2.1 mm, ID of 5 μm, Finnigan, Thermo, USA). Here, 20 μL of post-photocatalysis solution was injected automatically into the LC-MS system. The fluent contained 60% methanol and 40% water, and the flow rate was 0.2 mL min^−1^. MS conditions included an electrospray ionization interface, a capillary temperature of 27 °C with a voltage of 19.00 V, a spray voltage of 5,000 V and a constant sheath gas flow rate. The spectrum was acquired in the negative ion scan mode and the *m z^−1^* range swept from 50 to 600. Evolution of CO_2_ was analyzed with an Intersmat^TM^ IGC120-MB gas chromatograph equipped with a Porapack Q column (3 m in length and an inner diameter of 0.25 in.), which was connected to a catharometer detector. The total organic carbon (TOC) concentration was determined with a TOC analyzer (TOC-5000, Shimadzu Corporation, Japan). The photonic efficiency was calculated according to the following equation [43,44]:

*φ* = *R*/*I_o_* (7)

where *φ* was the photonic efficiency (%), *R* was the rate of MB degradation (Mol L^−1^ s^−1^), and *I_o_* was the incident photon flux (Einstein L^−1^ s^−1^).

## 4. Conclusions

The polyaniline-hybridized Bi_2_SnTiO_7_ was prepared for the first time by a chemical oxidation *in-situ* polymerization method and sol-gel method. The structural and photocatalytic properties of the polyaniline-hybridized Bi_2_SnTiO_7_ were investigated. XRD results indicated that Bi_2_SnTiO_7_ crystallized with the pyrochlore-type structure, cubic crystal system and space group *Fd3m*. The lattice parameter of Bi_2_SnTiO_7_ was found to be a = 10.52582(8) Å. Photocatalytic decomposition of aqueous MB was realized under visible light irradiation in the presence of the polyaniline-hybridized Bi_2_SnTiO_7_, Bi_2_InTaO_7_ or N-doped TiO_2_. The results showed that the polyaniline-hybridized Bi_2_SnTiO_7_ possessed higher catalytic activity compared with pure TiO_2_, Bi_2_InTaO_7_ or N-doped TiO_2_ for photocatalytic degradation of MB under visible light irradiation. The photocatalytic degradation of MB with the polyaniline-hybridized Bi_2_SnTiO_7_, Bi_2_InTaO_7_ or N-doped TiO_2_ as catalyst followed first-order reaction kinetics, and the first-order rate constant was 0.01504 min^−1^ or 0.00275 min^−1^ or 0.00333 min^−1^. Complete removal and mineralization of MB was observed after visible light irradiation for 220 minutes with the polyaniline-hybridized Bi_2_SnTiO_7_ as catalyst. The reduction of the total organic carbon, the formation of inorganic products such as SO_4_^2−^ and NO_3−_, and the evolution of CO_2_ revealed the continuous mineralization of MB during the photocatalytic process. The possible photocatalytic degradation pathway of MB was obtained under visible light irradiation. The polyaniline-hybridized Bi_2_SnTiO_7_/(visible light) photocatalysis system was found to be suitable for textile industry wastewater treatment and could be utilized to solve other environmental chemical pollution problems.
